# MicroRNA-410-3p attenuates gemcitabine resistance in pancreatic ductal adenocarcinoma by inhibiting HMGB1-mediated autophagy

**DOI:** 10.18632/oncotarget.22494

**Published:** 2017-11-18

**Authors:** Junjie Xiong, Dan Wang, Ailin Wei, Nengwen Ke, Yichao Wang, Jie Tang, Sirong He, Weiming Hu, Xubao Liu

**Affiliations:** ^1^ Department of Pancreatic Surgery, West China Hospital of Sichuan University, Chengdu 610041, China; ^2^ Department of Respiratory and Critical Care Medicine, West China Hospital of Sichuan University, Chengdu 610041, China; ^3^ Key Laboratory of Transplant Engineering and Immunology, Ministry of Health, Regenerative Medicine Research Center, West China Hospital, Sichuan University, Chengdu 610041, China; ^4^ Department of Thyroid Surgery, West China Hospital of Sichuan University, Chengdu 610041, China; ^5^ State Key Laboratory of Biotherapy, Sichuan University, Chengdu 610041, China; ^6^ Department of Immunology, College of Basic Medicine, Chongqing Medical University, Chongqing 400016, China

**Keywords:** PDAC, miR-410-3p, chemoresistance, HMGB1, autophagy

## Abstract

Gemcitabine-based chemotherapy is the most common treatment option for pancreatic ductal adenocarcinoma (PDAC). However, it offers little therapeutic value in many cases due to the rapid development of chemoresistance. MicroRNAs (miRNAs) have been found to play pivotal roles in the chemotherapeutic resistance of PDAC. We found that miR-410-3p was significantly down-regulated in human pancreatic cancer xenograft (HPCx) tumor tissues from gemcitabine-treated mice. Low miR-410-3p expression correlated with gemcitabine resistance in HPCx tumors and PDAC cells as well as poor prognosis in PDAC patients. We also found that miR-410-3p attenuated the gemcitabine resistance of PDAC by targeting the 3′-UTR of HMGB1. Moreover, our study clearly demonstrated that miR-410-3p enhanced chemosensitivity to gemcitabine via inhibiting HMGB1-induced autophagy during chemotherapy in PDAC cells. Our study suggests that miR-410-3p expression may be a useful indicator of the potential for chemoresistance to gemcitabine and provide a potential new therapeutic target for chemoresistance in PDAC.

## INTRODUCTION

Pancreatic ductal adenocarcinoma (PDAC) is one of the main causes of cancer-related death in developed countries and is considered to be an incurable and rapidly lethal disease [1]. The prognosis of patients after complete resection is poor, and >50% of patients develop tumor recurrence, with an estimated 5 year survival of only 20% [2]. Recent reports have suggested that adjuvant chemotherapy following curative surgery significantly prolonged the overall survival time after surgery, and this approach is being adopted as a standard strategy [3, 4]. The majority of PDACs are sensitive to gemcitabine at first [5], and gemcitabine-based chemotherapy has formed the core of adjuvant therapy for PDAC [3]. Unfortunately, gemcitabine is only modestly effective against tumor recurrence and extends overall survival (OS) by approximately 6 months. PDAC will eventually develop resistance to gemcitabine after prolonged exposure [6]. One of the most important factors affecting the poor prognosis of PDAC is considered to be its high resistance to most of the existing chemotherapeutic regimens.

MicroRNAs (miRNAs) are small non-coding RNAs that interact with mRNA and serve as negative regulators of gene expression by targeting the 3′-UTR mRNA region of the target mRNAs, inhibiting their translation or leading to their degradation [7, 8]. miRNAs have been found to play pivotal roles in PDAC development and progression by affecting multiple cellular processes, such as cell apoptosis, survival and chemotherapeutic resistance of PDAC [9, 10]. MiR-15a [11], miR-21 [12, 13], miR-34 [14], members of the miR-200 family [12, 15], miR-214 [11], miR-221 [16], members of the let7 family [15], and miR-320c [17] have been reported to play roles in gemcitabine chemoresistance in pancreatic cancer. Clinical studies have demonstrated the efficacy of miRNA as a therapeutic tool in the management of PDAC [18, 19]. Studies *in vitro* and *in vivo* have demonstrated the tremendous efficacy of miRNA as a therapeutic tool in the chemoresistance of PDAC to gemcitabine by regulating chemoresistance-related miRNAs. Down-regulation of miR-21 [20] and miR-125a [21] enhanced gemcitabine sensitivity in human pancreatic cancer cells. Up-regulation of miR-33a [22, 23], miRNA-181b [24] and miR-211 [25] increased the sensitivity of PDAC cells to gemcitabine *in vitro* and in nude mice.

In this study, we demonstrated that low miR-410-3p expression was associated with gemcitabine resistance in HPCx tumors and PDAC cells as well as poor prognosis in PDAC patients. Moreover, we found that up-regulated expression of miR-410-3p improved the chemoresistance of PDAC by targeting the 3′-UTR sequences of HMGB1 and inhibiting HMGB1-mediated autophagy signaling. As a result, this study provides a potential new strategy to enhance chemosensitivity of PDAC to gemcitabine.

## RESULTS

### Identification of chemoresistance-related miRNAs by miRNA microarray analysis

**Figure 1:**
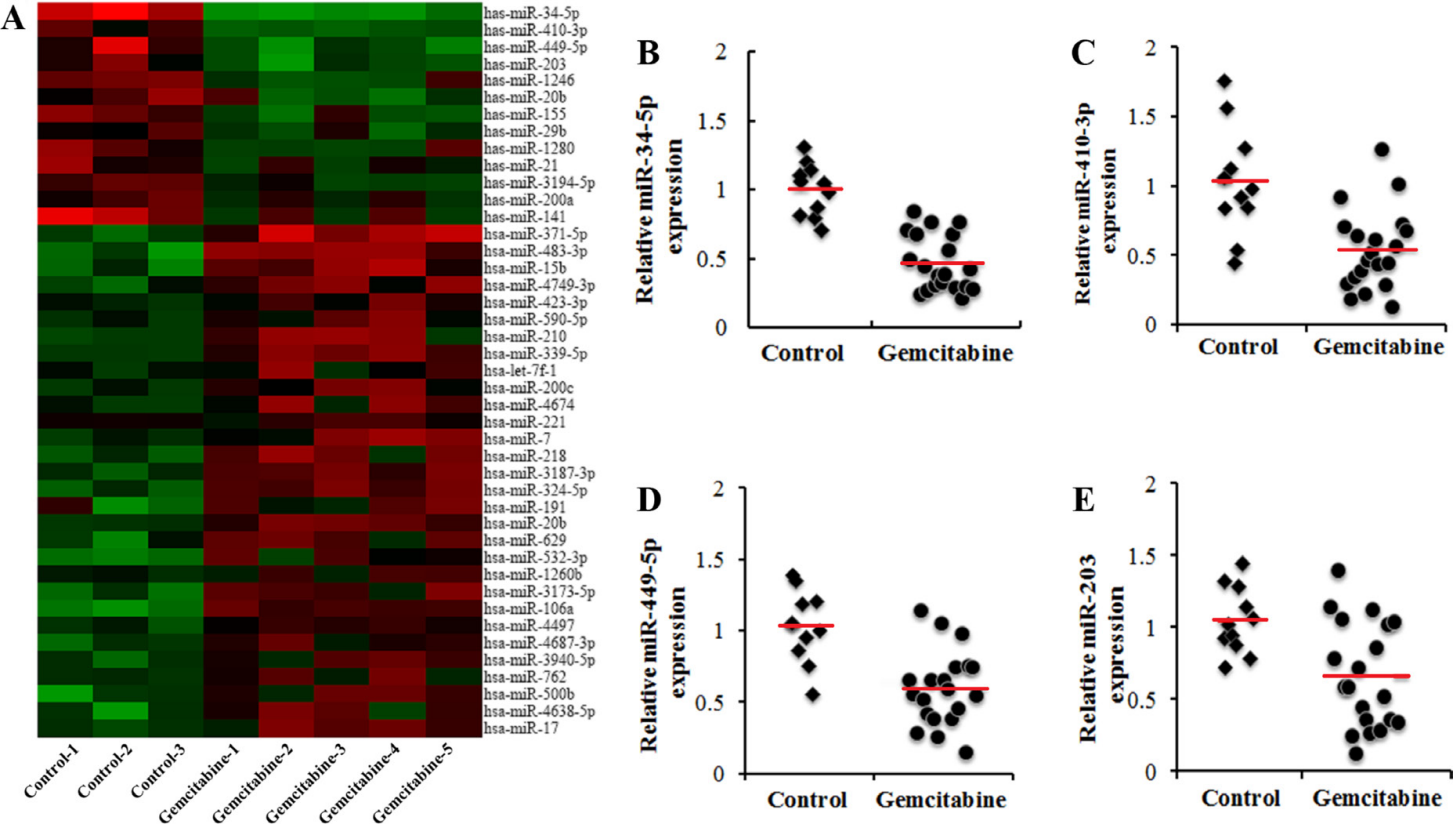
Identification of chemoresistance-related miRNAs in human pancreatic cancer xenografts (HPCx) tissues (**A**) miRNA microarray analysis of candidate miRNAs. The heat map revealed the miRNAs whose expression levels were altered >2 or <-2-fold in HPCx tumor tissues from gemcitabine-treated mice relative to expression in tissues from control mice (*n* = 11~20). miR-34-5p (**B**), miR-410-3p (**C**), miR-449-5p (**D**) and miR-203 (**E**) expression, determined by Real-time PCR, was down-regulated in HPCx tumor tissues from gemcitabine-treated mice (*p* < 0.05).

To identify potential miRNAs related to gemcitabine resistance in pancreatic cancer, we performed a miRNA microarray analysis in human pancreatic cancer xenograft (HPCx) subpopulations resistant to gemcitabine treatment. When the HPCx tumor volumes reached >80 mm^3^, mice were treated with gemcitabine, and the nodules were removed after 3 weeks. HPCx tumors from untreated mice were removed to serve as controls. Figure 1A shows that, among the 43 miRNAs whose expression levels were altered more than 1.5-fold, 13 candidate miRNAs were down-regulated in HPCx tumor tissues from gemcitabine-treated mice compared to their expression in controls tissues. Real-time PCR confirmed that miR-34-5p (Figure 1B), miR-410-3p (Figure 1C), miR-449-5p (Figure 1D) and miR-203 (Figure 1E) were down-regulated in HPCx tumor tissues from gemcitabine-treated mice (*P* < 0.05).

### miR-410-3p promotes the sensitivity of human PDAC to gemcitabine

**Figure 2:**
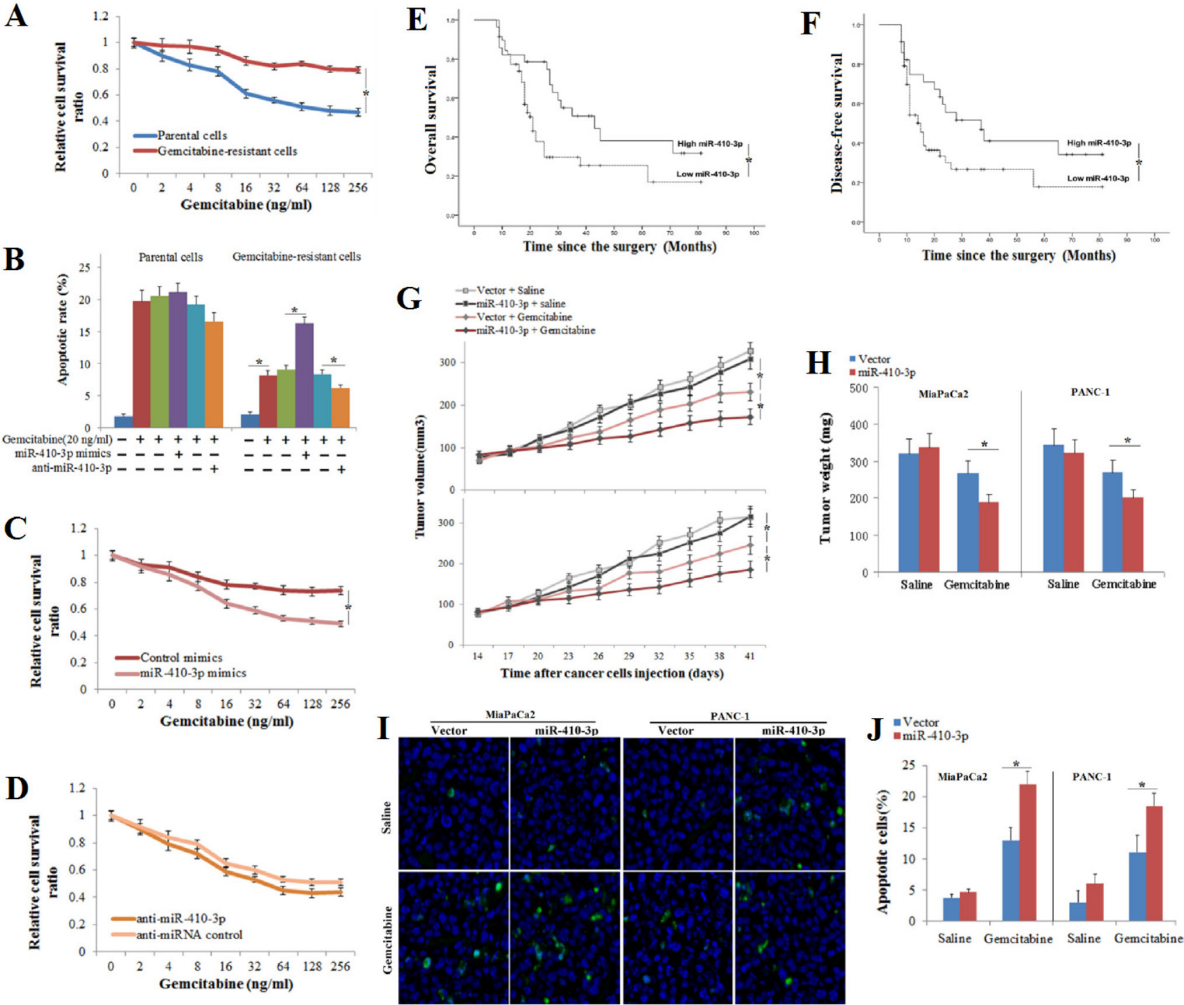
Association of miR-410-3p expression with the sensitivity to gemcitabine and the prognosis Growth-inhibitory effects (**A**) of gemcitabine on gemcitabine-resistant and parental clones of human PDAC cells were assessed by MTT assay. The apoptotic rate (**B**) of human PDAC cells (annexin V/PI double-positive cells) treated with 50 ng/ml gemcitabine were analyzed by FCM assay, and the growth-inhibitory effects of gemcitabine on gemcitabine-resistant clones of human PDAC cells treated with miR-410-3p mimics (**C**) and anti-miR-410-3p (**D**) were assessed by MTT assay. PDAC patients were divided into a low miR-410-3p expression group and high miR-410-3p expression group, according to the real-time PCR results. OS (**E**) and DFS (**F**) curves of PDAC patients with low or high miR-410-3p expression were drawn with the log-rank test. Human PDAC cells (MiaPaCa2 cell and PANC-1 cell) expressing miR-410-3p or a control vector were subcutaneously transplanted in mice. Mice were treated with gemcitabine or saline (*n* = 11~20). Tumor volume (**G**) and tumor weight (**H**) were measured. The number of apoptotic cells in the tumor specimens was determined using TUNEL staining (**I**). The TUNEL kit stained apoptotic cells green, while the nuclei were stained blue using DAPI. The rate of apoptosis (**J**) was determined by counting the TUNEL-stained cells in the specimen. All of the treatments in this figure were carried out in triplicate, and the results are displayed as the means ± SD. ^*^P < 0.05.

Gemcitabine-resistant clones of human PDAC cells were shown to be significantly more drug resistant to gemcitabine (Figure 2A, 2B). To evaluate the effect of miR-34-5p, miR-410-3p, miR-449-5p and miR-203 on the response to gemcitabine in human PDAC cells, mimics of these miRNAs were introduced into gemcitabine-resistant clones of human PDAC cells (Supplementary Figure 1). We found that up-regulation of miR-410-3p expression *significantly* inhibited chemoresistance to gemcitabine in human PDAC cells (Figure 2B, 2C). In contrast, the chemoresistance to gemcitabine was merely slightly repressed in human PDAC cells treated with miR-34-5p or miR-203 mimics (Supplementary Figure 2). Otherwise, anti-miR-410-3p transfection into human PDAC cells, whose endogenous levels of miR-410-3p were sufficiently inhibited (Supplementary Figure 1), resulted in significant augmentation of chemoresistance to gemcitabine in human PDAC cells (Figure 2B, 2D). We also examined the association between miR-410-3p expression and the clinicopathological features of 86 PDAC patients who received gemcitabine chemotherapy after radical surgical resection. There was no statistically significant association between miR-410-3p expression and the clinicopathological parameters (Supplementary Table 1). Kaplan-Meier survival analysis showed that PDAC patients with low miR-410-3p expression had a shorter overall survival (OS) time (Figure 2E, *P* = 0.018) and disease-free survival (DFS) time (Figure 2F, *P* = 0.037) than those with high miR-410-3p expression. These results demonstrated that low miR-410-3p expression correlated with gemcitabine resistance and poorer prognosis in human PDAC. To further confirm the function of miR-410-3p in gemcitabine sensitivity, we transplanted gemcitabine-resistant clones of human PDAC cells into mice that were then treated with gemcitabine or saline control. Overexpression of miR-410-3p significantly promoted the reduction in tumor volume (Figure 2G), tumor weight (Figure 2H), and apoptosis (Figure 2I, 2J) in tumors tissues caused by gemcitabine treatment. Taken together, these results confirmed that miR-410-3p attenuated the gemcitabine resistance of PDAC *in vivo and vitro.*

### Predicting and validating the gene targets of miR-410-3p that affected gemcitabine resistance

**Figure 3:**
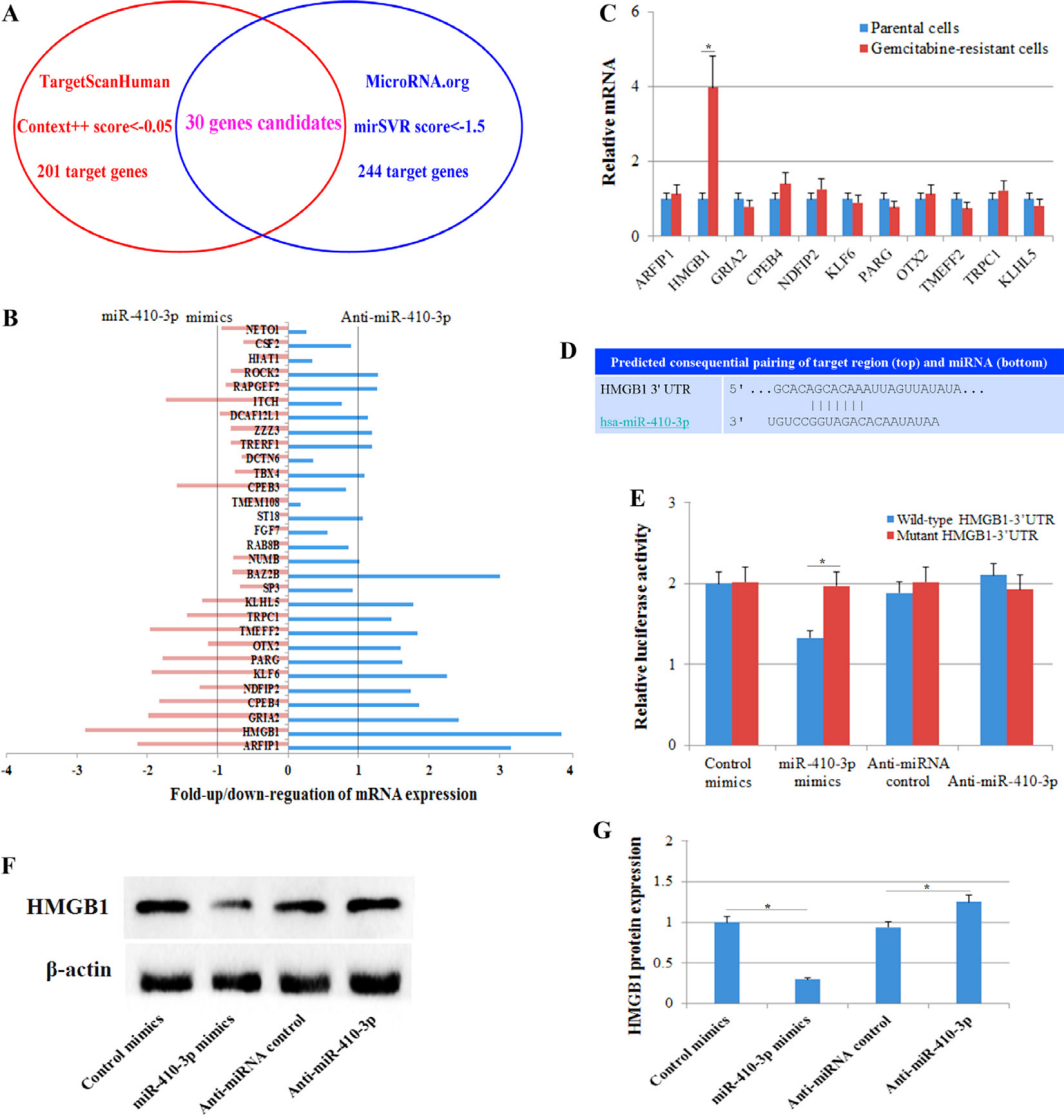
Predicting and validating the gene targets of miR-410-3p (**A**) Online programs (TargetScanHuman 7.1 and MicroRNA.org) predicted putative gemcitabine resistance-related target genes of miR-410-3p. TargetScanHuman identified 201 target genes (Context++ score < –0.05), and MicroRNA.org identified 244 target genes (mirSVR score < –1.5). Thirty gene candidates were designated putative target genes of miR-410-3p by both programs. (**B**) The expression of the 30 putative target genes was determined by real-time PCR in human PDAC cells transfected with miR-410-3p mimics or anti-miR-410-3p. (**C**) The expression of 11 potential targets (ARFIP1, HMGB1, GRIA2, CPEB4, NDFIP2, KLF6, PARG, OTX2, TMEFF2, TRPC1 and KLHL5) was determined by real-time PCR in gemcitabine-resistant and parental clones of human PDAC cells. (**D**) Predicted miR-410-3p binding sites in the 3′-UTR of HMGB1. (**E**) Luciferase reporter assay with co-transfection of wild-type or mutant HMGB1 and miR-410-3p mimics, anti-miR-410-3p, mimics-control or anti-miRNA-control in human PDAC cells. (**F**, **G**) Western blot analysis of HMGB1 expression in gemcitabine-resistant clones of human PDAC cells transfected with miR-410-3p mimics, anti-miR-410-3p, mimics-control or anti-miRNA-control. All of the treatments in this figure were carried out in triplicate, and the results are displayed as the means ± SD. ^*^P < 0.05.

To examine in more detail the function of miR-410-3p in the sensitivity of pancreatic cancer to gemcitabine treatment, we used online programs (TargetScanHuman 7.1 and MicroRNA.org) to search for potential targets of miR-410-3p and to identify those putative targets that might be related to chemosensitivity. Thirty gene candidates were found to be potential targets of miR-410-3p by both programs (Figure 3A, Table 1). We analyzed the expression of the 30 putative target genes after modification of the expression of miR-410-3p in human PDAC cells via up/down-regulation of miR-410-3p expression by transfection with miR-410-3p mimics or anti-miR-410-3p oligonucleotide, respectively. Eleven potential targets (ARFIP1, HMGB1, GRIA2, CPEB4, NDFIP2, KLF6, PARG, OTX2, TMEFF2, TRPC1 and KLHL5) were significantly affected by the miR-410-3p expression modifications (Figure 3B). By examining these 11 potential targets, we further found that only HMGB1 was significantly expressed in gemcitabine-resistant PDAC cells (Figure 3C), indicating that HMGB1 might be related to chemosensitivity. A luciferase assay was performed to further confirm that miR-410-3p can directly target the 3′-UTR of HMGB1 (Figure 3D, 3E). Figure 3F illustrates that HMGB1 was shown to be down-regulated by miR-410-3p overexpression and up-regulated by miR-410-3p silencing at the protein level in human PDAC cells (Figure 3G). These results showed that HMGB1 was a direct target of miR-410-3p and might be related to gemcitabine resistance in human PDAC cells.

**Table 1:** Predicted miR-410-3p targets & target downregulation scores

Ortholog of target gene	Gene name	3P-seq tags + 5	mirSVR score	Cumulative weighted context++ score	Total context++ score	Previous TargetScan publication (s)
BAZ2B	bromodomain adjacent to zinc finger domain, 2B	25	–3.84	–0.11	–0.13	2011
DCAF12L1	DDB1 and CUL4 associated factor 12-like 1	5	–3.15	–0.23	–0.23	2011
ARFIP1	ADP-ribosylation factor interacting protein 1	275	–2.9	–0.34	–0.34	
HMGB1	high mobility group box 1	800	–2.64	–0.25	–0.32	2007, 2009, 2011
GRIA2	glutamate receptor, ionotropic, AMPA 2	5	–2.64	–0.08	–0.08	2007, 2009, 2011
CPEB4	cytoplasmic polyadenylation element binding protein 4	472	–2.55	–0.09	–0.09	2007, 2009, 2011
ITCH	itchy E3 ubiquitin protein ligase	769	–2.42	–0.11	–0.13	2011
RAPGEF2	Rap guanine nucleotide exchange factor (GEF) 2	484	–2.33	–0.14	–0.14	2009, 2011
ROCK2	Rho-associated, coiled-coil containing protein kinase 2	142	–2.2	–0.05	–0.07	
NDFIP2	Nedd4 family interacting protein 2	1747	–2.01	–0.18	–0.3	2007, 2011
ZZZ3	zinc finger, ZZ-type containing 3	338	–2	–0.3	–0.42	2007, 2009, 2011
KLF6	Kruppel-like factor 6	4644	–2	–0.14	–0.14	2011
PARG	poly (ADP-ribose) glycohydrolase	445	–1.99	–0.15	–0.21	
DCTN6	dynactin 6	745	–1.94	–0.48	–0.5	2009, 2011
TRERF1	transcriptional regulating factor 1	52	–1.93	–0.07	–0.07	2011
OTX2	orthodenticle homeobox 2	5	–1.92	–0.28	–0.28	2007, 2011
CPEB3	cytoplasmic polyadenylation element binding protein 3	48	–1.92	–0.06	–0.06	2007, 2009, 2011
TBX4	T-box 4	21	–1.89	–0.1	–0.1	
TMEFF2	transmembrane protein with EGF-like and two follistatin-like domains 2	179	–1.84	–0.13	–0.39	2007, 2009, 2011
TMEM108	transmembrane protein 108	31	–1.84	–0.08	–0.15	2011
TRPC1	transient receptor potential cation channel, subfamily C, member 1	18	–1.81	–0.23	–0.23	2009, 2011
SP3	Sp3 transcription factor	285	–1.79	–0.11	–0.12	2007, 2009, 2011
NUMB	numb homolog (Drosophila)	2707	–1.72	–0.17	–0.18	2007, 2009, 2011
RAB8B	RAB8B, member RAS oncogene family	45	–1.7	–0.07	–0.09	2007, 2009, 2011
FGF7	fibroblast growth factor 7	5	–1.68	–0.09	–0.09	2007, 2009, 2011
ST18	suppression of tumorigenicity 18 (breast carcinoma) (zinc finger protein)	5	–1.61	–0.05	–0.05	2007, 2009, 2011
KLHL5	kelch-like family member 5	366	–1.59	–0.12	–0.18	2011
HIAT1	hippocampus abundant transcript 1	1473	–1.56	–0.16	–0.17	2007
CSF2	colony stimulating factor 2 (granulocyte-macrophage)	103	–1.51	–0.16	–0.16	2007, 2009, 2011
NETO1	neuropilin (NRP) and tolloid (TLL)-like 1	5	–1.5	–0.21	–0.24	2011

### Down-regulation of HMGB1 is essential for the miR-410-3p-attenuated gemcitabine resistance in human PDAC cells

**Figure 4:**
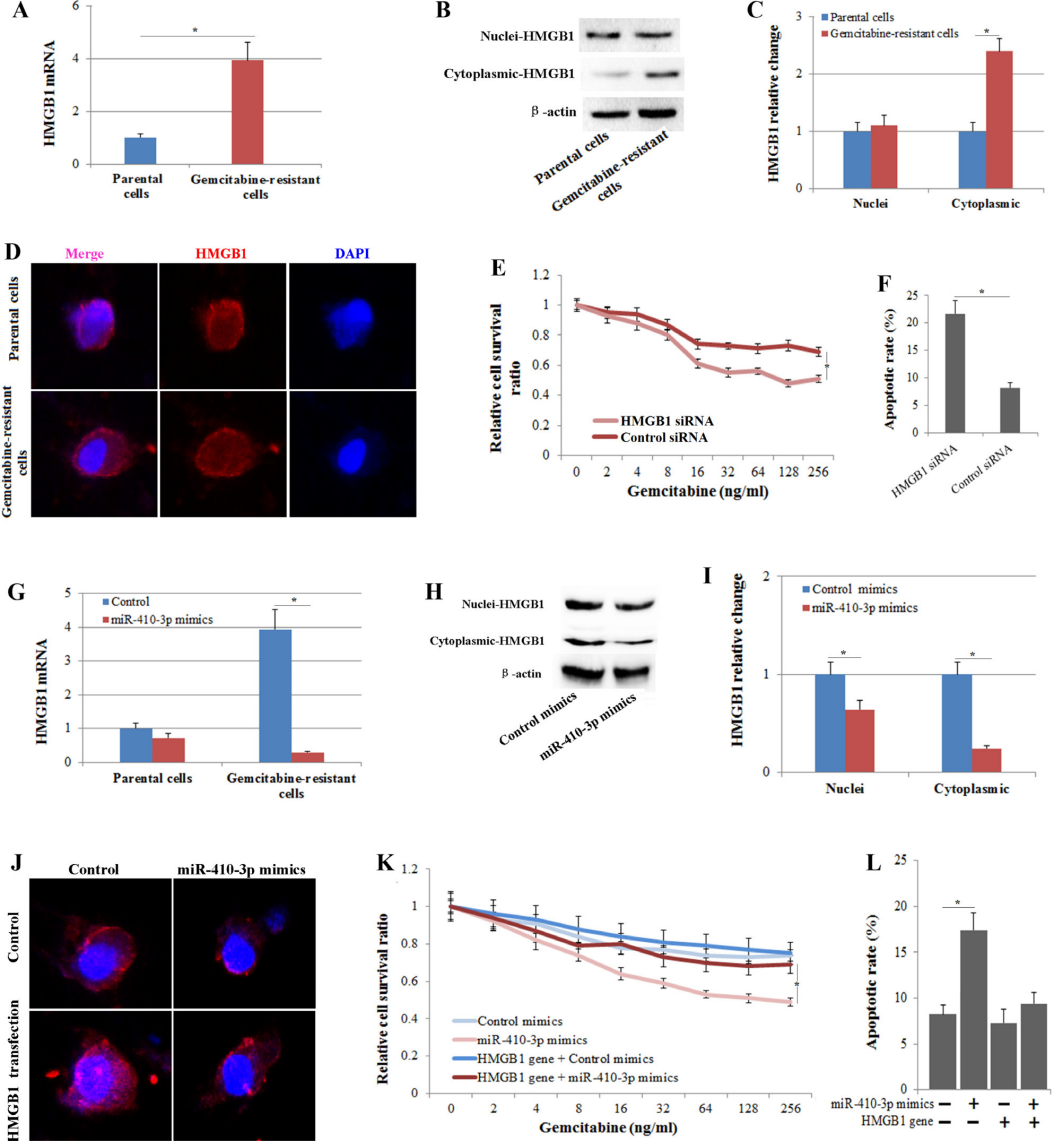
The expression and translocation of HMGB1 correlates with gemcitabine resistance in human PDAC cells HMGB1 expression was determined by real-time PCR (**A**), Western blot (**B**) and immunofluorescence (**D**) in gemcitabine-resistant and parental clones of human PDAC cells. (**C**) The relative change in nuclear and cytoplasmic HMGB1 expression was quantified in gemcitabine-resistant clones of human PDAC cells relative to that in parental clones.Growth-inhibitory effects (**E**) and the apoptotic rate (annexin V/PI double-positive cells) (**F**) of gemcitabine on gemcitabine-resistant PDAC cells treated with or without HMGB1 siRNA were assessed by MTT assay and FCM assay, respectively. (**G**) HMGB1 expression was determined by real-time PCR in gemcitabine-resistant and parental clones of human PDAC cells treated with or without miR-410-3p mimics. HMGB1 expression was determined by Western blot (**H**) and immunofluorescence (**J**) in gemcitabine-resistant clones of human PDAC cells treated with or without miR-410-3p mimics and/ or HMGB1 gene transfection. The relative change in nuclear and cytoplasmic HMGB1 expression (**I**) was quantified relative to that in clones treated with mimics-control. Growth-inhibitory effects (**K**) and the apoptotic rate (annexin V/PI double-positive cells) (**L**) of gemcitabine on gemcitabine-resistant PDAC cells treated with or without miR-410-3p mimics and/or HMGB1 gene transfection were assessed by MTT assay and FCM assay, respectively. All of the treatments in this figure were carried out in triplicate, and the results are displayed as the means ± SD. ^*^P < 0.05.

As a nuclear protein, the expression and translocation of HMGB1 plays a critical role in the response to chemotherapy in cancer [26]. Therefore, we examined whether the expression and translocation of HMGB1 correlated with gemcitabine resistance in human PDAC cells. The level of HMGB1 mRNA was significantly increased in gemcitabine-resistant clones of human PDAC cells (Figure 4A). Western blot (Figure 4B, 4C) and immunofluorescence (Figure 4D) analysis indicated a prominent cytoplasmic expression of HMGB1 in gemcitabine-resistant PDAC cells. Moreover, we transfected gemcitabine-resistant PDAC cells with HMGB1 siRNA (Figure 4A) and found that HMGB1 silencing significantly inhibited gemcitabine resistance (Figure 4E, 4F) to a level similar to that of cells treated with miR-410-3p mimics. To further investigate whether HMGB1 was responsible for the miR-410-3p-attenuated gemcitabine resistance in human PDAC cells, gemcitabine-resistant PDAC cells were treated with miR-410-3p mimics and/or HMGB1 gene transfection. Exogenous miR-410-3p significantly reduced the expression and translocation (Figure 4G–4J) of HMGB1 and increased the gemcitabine chemosensitivity (Figure 4K, 4L) of human PDAC cells. More important, when the expression of HMGB1 was increased by HMGB1 gene transfection (Figure 4J), miR-410-3p treatment did not repress the gemcitabine resistance in human PDAC cells (Figure 4K, 4L). Thus, HMGB1 mediates the effects of miR-410-3p on the chemosensitivity of human PDAC cells to gemcitabine.

### MiR-410-3p inhibits HMGB1-mediated autophagy in human PDAC cells during chemotherapy

**Figure 5:**
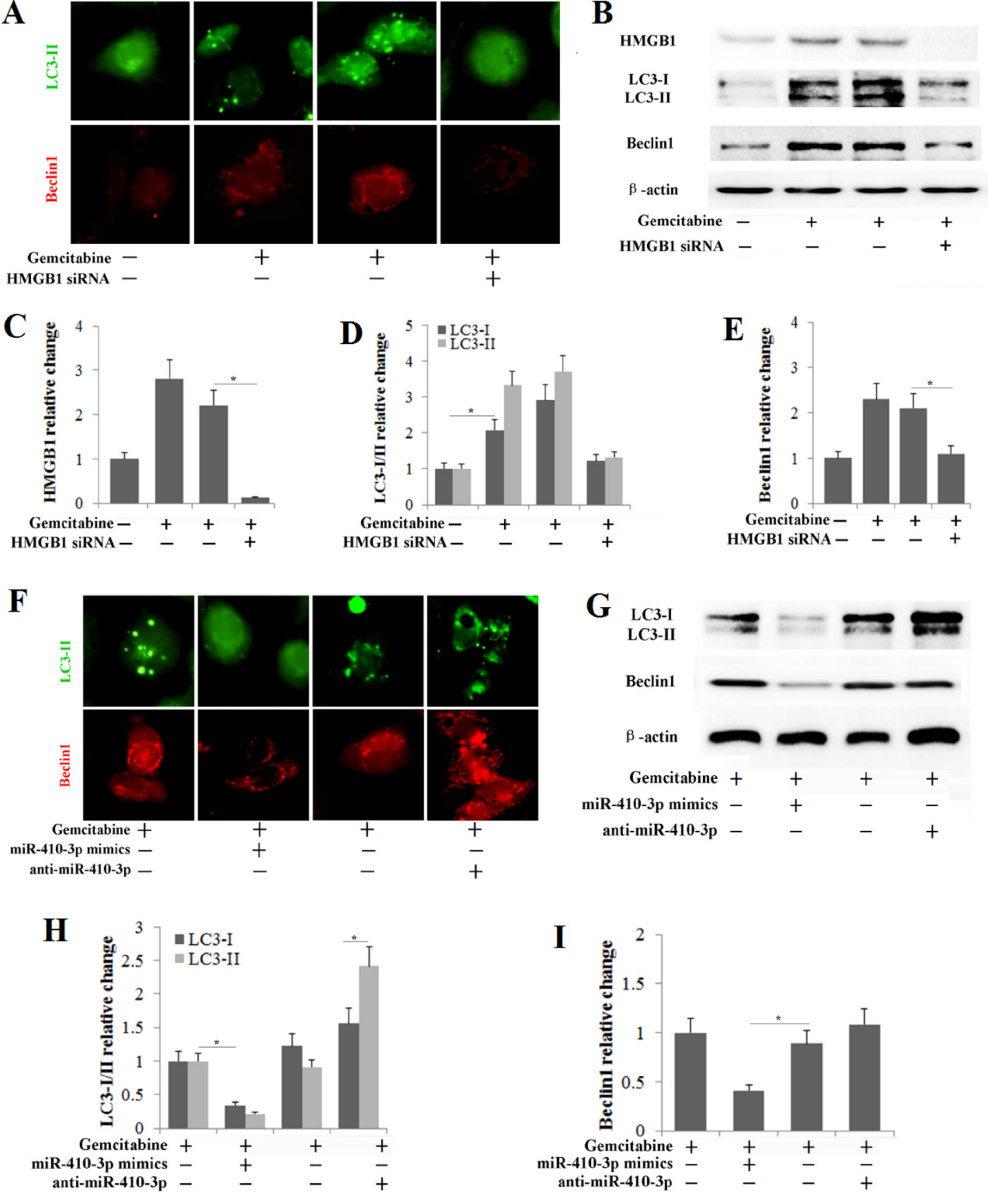
miR-410-3p inhibits HMGB1-mediated autophagy in human PDAC cells during chemotherapy (**A**) Human PDAC cells that stably express GFP-LC3 were used to detect autophagy (Tip), and immunofluorescence was used to determine Beclin1 expression (Sif) after the PDAC cells were treated with or without 50 ng/ml gemcitabine and/or HMGB1 siRNA. (**B**) HMGB1, LC3 and Beclin1 expression levels were determined by Western blot in gemcitabine-resistant PDAC cells treated with or without 50 ng/ml gemcitabine and/or HMGB1 siRNA. The relative change in HMGB1 (**C**), LC3-I/II (**D**) and Beclin1 (**E**) was quantified relative to that in untreated controls. (**F**) Human PDAC cells that stably express GFP-LC3 were used to detect autophagy (Tip), and immunofluorescence was used to determine Beclin1 expression (Sif) after the gemcitabine-resistant cells were treated with 50 ng/ml gemcitabine and/or miR-410-3p mimics or anti-miR-410-3p. (**G**) LC3 and Beclin1 expression levels were determined by Western blot in gemcitabine-resistant PDAC cells treated with 50 ng/ml gemcitabine and/or miR-410-3p mimics or anti-miR-410-3p. The change in LC3-I/II (**H**) and Beclin1 (**I**) expression was quantified relative to that in gemcitabine-treated controls. All of the treatments in this figure were carried out in triplicate, and the results are displayed as the means ± SD. ^*^P < 0.05.

Tumor cells use cytoprotective autophagy as a defense from apoptotic cell death, which in turn contributes to the development of chemoresistance [27, 28]. To investigate whether gemcitabine treatment induced autophagy in human PDAC cells, cells were treated with 50 ng/ml of gemcitabine for 48 h as in previous studies [29, 30]. In our study, chemotherapy-stimulated autophagy in human PDAC cells was also confirmed by assays examining the expression of autophagy-related biomarkers. The number of GFP-LC3 fluorescent dots and the accumulation of LC3 puncta in PDAC cells that stably expressed GFP-LC3 was significantly higher in the gemcitabine treatment groups (Figure 5A), and significantly high levels of LC3-I/-II conversion and Beclin1 expression were also observed in PDAC cells treated with gemcitabine (Figure 5B, 5D, 5E). The interaction of HMGB1 with Beclin1 and LC3 plays a role in the facilitation of autophagy following cytotoxic insults [31–33]. Thus, we further determined the correlation of the high autophagy level following chemotherapy with the up-regulation of HMGB1 in human PDAC cells. We knocked out HMGB1 in human PDAC cells (Figure 5B, 5C) and then treated them with gemcitabine. As expected, HMGB1 knockdown in gemcitabine-treated cells decreased the levels of autophagy as shown by the decrease in LC3 punctae, LC3-I/II conversion and Beclin1 expression (Figure 5A, 5B, 5D, 5E). These results confirmed that HMGB1 mediated autophagy during chemotherapy in PDAC cells. Our studies had previously shown that HMGB1 was a direct target of miR-410-3p in PDAC cells; therefore, we investigated this effect further to determine whether miR-410-3p could suppress HMGB1-mediated autophagy in human PDAC cells during chemotherapy. MiR-410-3p overexpression significantly decreased LC3 punctae, LC3-I/II conversion and Beclin1 expression, while miR-410-3p silencing increased LC3 punctae, LC3-I/II conversion and Beclin1 expression (Figure 5F–5I). These results revealed that overexpression of miR-410-3p can inhibit HMGB1-mediated autophagy in human PDAC cells.

## DISCUSSION

Chemotherapy is the most common treatment option for many cancers, including pancreatic cancer. However, it offers little therapeutic value in many cases due to the rapid development of chemoresistance. Recently, several studies have indicated that some miRNAs [34], such as miR-21 [35], miR-155 [36], miR-320c [18] and miR-1246 [37], have been involved in the induction of chemoresistance in PDAC. The identification of key miRNA networks in pancreatic cancer will provide long-awaited diagnostic, therapeutic, and prognostic tools for early detection, better treatment options, extended life expectancy, and better quality of life in PDAC patients [38]. Thus, we identified potential miRNAs related to gemcitabine resistance in a human pancreatic cancer xenograft (HPCx) with miRNA microarray analysis and showed that miR-34-5p, miR-410-3p, miR-449-5p and miR-203 were significantly down-regulated in HPCx tumor tissues from gemcitabine-treated mice. In addition, we found that low miR-410-3p expression correlated with gemcitabine resistance and poorer prognosis in human PDAC.

The most widely used chemotherapeutic treatment for pancreatic cancer is gemcitabine, which shows a moderate tumor suppression response rate of ~12% [18]. Therefore, intense research efforts are required to improve the outcome for successful pancreatic cancer treatment. Studies *in vitro* and *in vivo* have demonstrated the tremendous efficacy of miRNA as a therapeutic tool in the chemoresistance of PDAC to gemcitabine by regulating chemoresistance-related miRNAs. Down-regulation of miR-21 [20] and miR-125a [21] enhanced gemcitabine sensitivity in human pancreatic cancer cells. Up-regulation of miR-33a [22, 23], miRNA-181b [24] and miR-211 [25] increased the sensitivity of PDAC cells to gemcitabine *in vitro* and in nude mice. Evidence has shown that miR-410-3p acts as an oncogene or tumor-suppressor gene in various types of cancer, including cholangiocarcinoma [39], colorectal cancer [40], breast cancer [41] and pancreatic cancer [42]. Re-expression of miR-410 enhanced the sensitivity of adjuvant radiotherapy in gastric cancer cells [43]. Here, we identified miR-410-3p as a potential therapeutic target of chemoresistance to gemcitabine in PDAC. The *in vitro* MTT assay and FCM apoptosis assay after up-regulation of miR-410-3p in PDAC cells suggested that miR-410-3p up-regulation is sufficient to enhance the growth-inhibitory effects and apoptosis caused by gemcitabine treatment. Overexpression of miR-410-3p promoted the reduction in the volume, weight, and apoptosis of HPCx tumor tissues caused by gemcitabine treatment *in vivo*. Our result is consistent with some reports that have identified miR-410-3p as a potential therapeutic target of chemoresistance to gemcitabine in PDAC.

A better understanding of disease pathogenesis mechanisms will facilitate the early detection and development of effective treatments. We used online programs to search for the potential targets of miR-410-3p and found that HMGB1 was significantly affected by both up- and down-regulation of miR-410-3p expression and expressed in gemcitabine-resistant PDAC cells. Moreover, a luciferase assay confirmed that miR-410-3p can directly target the 3′-UTR sequences of HMGB1. Consistent with the dual luciferase assay, ChIP analysis also observed the direct interaction between miR-410-3p and HMGB1 (Supplementary Figure 3A). In addition, up/down-regulation of miR-410-3p expression by transfection with miR-410-3p mimics or anti-miR-410-3p did not affect the expression of HMGB2, HMGB3 or HMGB4 in human PDAC cells (Supplementary Figure 3B). HMGB1, known primarily as a regulator of autophagy, is expressed in various normal cells and has also been confirmed to be involved in cancer development by interfering with several signaling pathways [44, 45]. Recently, studies have suggested that HMGB1 binds to the autophagy regulator Beclin1 and regulates the formation of the Beclin1-PI3KC3 complex that facilitates autophagic progression [31]. Induction of autophagy by HMGB1 is important for tumor development and a novel target for cancer therapy [31, 46]. Autophagy is a natural destructive mechanism of cells and is characterized as a degrading and recycling of cellular components for new cell formation [27]. Tumor cells use cytoprotective autophagy as a defense against apoptosis cell death, which in turn contributes to the development of chemoresistance. There is mounting evidence that autophagy contributes to increased gemcitabine resistance in PDAC cancer [28, 47, 48].

Some studies have also reported that miRNAs can modulate chemotherapy via autophagy [49, 50]. MicroRNA-410 enhances chemosensitivity via regulation of autophagy-related genes [51]. However, the regulatory mechanisms by which miR-410-3p effects chemoresistance of PDAC to gemcitabine remain largely unknown. The present study revealed that miR-410-3p attenuated gemcitabine resistance in PDAC cancer cells by targeting the 3′UTR of HMGB1. HMGB1-mediated autophagy during chemotherapy in PDAC cells was further confirmed by autophagy-related biomarker assays. Autophagy was inhibited after transfection of PDAC cells with HMGB1 siRNA or miR-410-3p mimics during chemotherapy. Further investigations indicated that cell viability was significantly decreased and apoptosis was significantly increased in the gemcitabine-treated PDAC cells transfected with miR-410-3p mimics. Conversely, miR-410-3p silencing was able to effectively induce autophagic activation, promote cell growth and suppress cell apoptosis in the gemcitabine-treated PDAC cells. Taken together, our results of the present study provided novel evidence suggesting that miR-410-3p attenuates chemoresistance of PDAC to gemcitabine by inhibiting HMGB1-induced autophagy.

In summary, by using miRNA microarray and RT-PCR analysis, we demonstrated that miR-410-3p was down-regulated in HPCx tumor tissues from gemcitabine-treated mice and found that low miR-410-3p expression was associated with gemcitabine resistance in HPCx tumors and PDAC cells as well as poor prognosis in PDAC patients. On the other hand, both *in vivo* and *in vitro*, our study demonstrated that overexpression of miR-410-3p attenuated gemcitabine resistance of PDAC by targeting the 3′-UTR sequences of HMGB1 and inhibiting HMGB1-mediated autophagy. Together, our studies suggest that miR-410-3p expression may be a useful indicator of the potential for chemoresistance to gemcitabine and provide a potential new therapeutic target for chemoresistance in PDAC.

## MATERIALS AND METHODS

### Cell culture and treatment

Human PDAC cell lines (MiaPaCa2 and PANC-1, Shanghai Cell Bank, China) were cultured in DMEM medium [23] (Invitrogen, CA) supplemented with 10% fetal bovine serum, 0.5 mM sodium pyruvate (Sigma-Aldrich, Steinheim, Germany), 50 units/ml penicillin, and 50 μg/ml streptomycin. The cells were maintained in a 5% CO2-humidified atmosphere at 37°C. Gemcitabine-resistant cells were generated by exposure to gradually increasing concentrations of the drug for 2 months. Parental MiaPaCa2 cells were exposed to gemcitabine (Eli Lilly Pharmaceuticals, Indianapolis, USA) at an initial concentration of 1 ng/ml. When cells adapted to the drug, the gemcitabine concentration was gradually increased to the final concentrations (20 ng/ml).

Cells were transfected with miR-410-3p mimics or miRNA scrambled controls at a final concentration of 20 nM using Oligofectamine (Invitrogen), or the antisense oligonucleotide inhibitor of hsa-miR-155 (anti-miR-410-3p) or their scrambled oligonucleotides (anti-miRNA control) at a final concentration of 200 nM using the DharmaFECT3 transfection reagent (Dharmacon), or with the empty vector pEGFP-N1 or the human pEGFP-N1-HMGB1 vector using the ExGen-500 transfection reagent (Fermentas, Germany), or with 100 nmol/l human HMGB1 small interfering (si)RNA or a control siRNA (Santa Cruz) using RNAi MAX transfection reagent. In apoptosis experiments, the PDAC cells treated with 50 ng/ml gemcitabine or PBS for 72 h before flow cytometry assay.

Growth inhibition assay and the dual-parameter (PI/ Annexin V) flow cytometry were performed to detect the cell viability and apoptosis. Real-time PCR was used to detect the expression levels of miR-34-5p, miR-410-3p, miR-449-5p, miR-203, HMGB1, ARFIP1, GRIA2, CPEB4, NDFIP2, KLF6, PARG, OTX2, TMEFF2, TRPC1 and KLHL5. Western blotting was used to detect the expression of HMGB1, LC3 and Beclin1. Immunofluorescence staining was performed to detect the expression of HMGB1 and Beclin1.

### *In vivo* experiments

This study was approved by the Animal Experiments Committee, West China Hospital, Sichuan University. It was performed in accordance with the National Institutes of Health guidelines for the use of experimental animals. Six-week-old female nude mice were purchased from Dossy Biological Technology Co. Ltd. (Chengdu, China), and maintained in a specific pathogen-free environment. For the human pancreatic cancer xenografts (HPCx), MiaPaCa2 or PANC-1 cells (1 × 10^6^) were subcutaneously transplanted into the interscapular fat pad of female nude mice under anesthesia. The mice were administered gemcitabine (125 mg/kg) intraperitoneally three times on days 17, 24, and 31 after xenograft transplantation. Subcutaneous tumor volume was calculated as follows: (greatest diameter) × (shortest diameter)^2^ × 0.5. Gemcitabine therapy was initiated when the tumor volume was 80~100 mm^3^. Mice were euthanized on day 41. The tumor tissue sections were stained with hematoxylin and eosin; alternatively, a TUNEL assay was performed on the sections using an *in situ* cell death detection kit (Roche Diagnostics). MicroRNAs were identified by miRNA microarray analysis (Agilent Technologies) in tumor tissues from treated mice with gemcitabine relative to from controls mice.

### Clinical samples and data

86 PDAC in patients who received gemcitabine chemotherapy after radical surgical resection were collected between July 2010 and March 2014 at the West China Hospital of Sichuan University. Gemcitabine chemotherapy was started within 8 weeks postresection. Gemcitabine (intravenous infusion of 1000 mg/m^2^) was given on days 1, 8, and 15 of each cycle, to be repeated every 4 weeks. This lasted for 6 months. Written informed consent was obtained from all patients involved, and the PDAC diagnosis was based on hematoxylin eosin and immunohistochemical staining from tumor tissue sections. None of the patients received any other preoperative and postoperative chemotherapy or radiotherapy. The clinical data were collected from each patient, including age, gender, location, tumor size and lymph node metastasis, degree of differentiation, pT category, the presence of metastatic disease, and survival. Our study received approval from Institutional Review Board of Sichuan University West China Hospital. Patients were followed up till the end of April 2017, either in the form of telephonic conversation or as outpatient clinic appointment. Real-time PCR was used to detect the expression levels of miR-410-3p in clinical PDAC samples.

### Statistical analysis

The results presented are the average of at least three experiments, each performed in triplicate with standard errors. Kruskal-Wallis H test and Chi-square test were used to analyse the expression rate in all groups. A Kaplan-Meier survival analysis was performed to assess the OS and DFS of the patients. One-way analysis of variance (ANOVA) was used to analyze the differences between groups. Statistical analyses were conducted by SPSS 20.0, and a *P*-value less than 0.05 was considered to be statistically significant.

Supplemental Information includes Extended Experimental Procedures

## SUPPLEMENTARY MATERIALS FIGURES AND TABLES


