# Supplementary material for: MicroRNA-410-3p attenuates gemcitabine resistance in pancreatic ductal adenocarcinoma by inhibiting HMGB1-mediated autophagy
Source: Oncotarget. 2017 Nov 18;8(64):107500–12. doi: 10.18632/oncotarget.22494 (PMC5746084; doi:10.18632/oncotarget.22494)
Supplement: Supplementary file 1 [file oncotarget-08-107500-s001.pdf]

MicroRNA-410-3p attenuates gemcitabine resistance in pancreatic ductal adenocarcinoma by inhibiting HMGB1-mediated autophagy

SUPPLEMENTARY MATERIALS

Growth inhibition assay and determination of cell viability

Growth inhibition was assessed using the 3-(4,5-dimethylthiazol-2-yl)-2,5-diphenyl tetrazolium bromide (MTT; Sigma-Aldrich Co.). In brief, cells were incubated for 72 hours under several concentrations of gemcitabine, and then cell viability was evaluated by absorbance using MTT solution. The results were expressed as the percentage of absorbance relative to that of untreated controls.

Determination of cell apoptosis

The cell were incubated with propidium iodide (PI) and annexin V-FITC (annexin V). The dual-parameter (PI/Annexin V) flow cytometry was used to determine the number of apoptotic cells (annexinV/PI double-positive cells).

MiRNA microarray

Total RNA samples were spiked using the MicroRNA Spike-In Kit (Agilent Technologies) to assess the labeling and hybridization efficiencies. The miRNAs microarray data were normalized using the GeneSpring GX software version 12.0 (Agilent Technologies). The signal values were transformed to the log base 10, and then quantile and percentile shift was applied to obtain an equal distribution of probe signal intensities. Compared with the expression level of the reference RNA, the miRNAs were described as differentially expressed if the p-values were < 0.05, and the fold change (FC) was greater than 2 or less than 0.5.

Real-time PCR

RNA was extracted using Trizol (Invitrogen) or miRVANA (Ambion) kits following the manufacturer's instruction. The quantitative analysis of the change in expression levels was calculated by the real-time PCR machine (iQ5, Bio-Rad). The reactions were performed in duplicate, and the delta-delta-Cycle Threshold (ddCt) values were calculated on the basis of the average of the normalization genes, and the results were normalized to the average of the results obtained for GAPDH or RNU6B.

Western blot analysis

Protein expression levels were assessed using Western blot analysis. In brief, the total cell lysates were separated on SDS-PAGE gels, transferred to PVDF membranes (Millipore), immunoblotted with antibodies, and visualized using an enhanced chemiluminescence detection system (Amersham Biosciences). The protein bands were quantitated by densitometry using gel analysis software Image J (rsbweb.nih.gov/ij).

Dual luciferase reporter assay

The fragment from HMGB1-3′-UTR containing the predicted miR-410-3p binding site was amplified by PCR and then cloned into a pmirGLO Dual-Luciferase miRNA Target Expression Vector (Promega) to form the reporter vector HMGB1-3′-UTR wild type. The putative binding site of miR-410-3p in the HMGB1-3′-UTR was mutated by using a site-directed mutagenesis kit from Fast Mutagenesis System (TransGen Biotech, Beijing, China), and the mutant reporter vector was named as HMGB1-3′ -UTR mutant. The miR-410-3p mimic and vector were co-transfected into human PDAC cells, and Renilla luciferase reporter plasmid (pRL-TK) was also co-transfected as the internal reference. After transfection for 48 h, cells were lysed in passive lysing buffer, and then firefly and Renilla luciferase activities were analyzed using the Dual-Luciferase Reporter Assay System (Promega). The results of firefly luciferase activity were normalized to the Renilla luciferase activity.

Detection of green fluorescent protein (GFP)-LC3 autophagic dots

Cell strains that stably expressed GFP-LC3 were generated by transfection of cells with pcDNA3.1-GFP-LC3 vectors. GFP-LC3 stably expressed high levels of the GFP-LC3 protein. The GFP-LC3 stably expressing cells were detected and confirmed by fluorescence microscopy,

and used for the following experiments. The cytosolic form of LC3 (LC3-I) is conjugated to phosphatidylethanolamine to form the LC3-phosphatidylethanolamine conjugate (LC3-II), which is recruited to autophagosomal membranes and reflects autophagic activity. The GFP-LC3 expressing cells were subsequently exposed to the drugs, and autophagosomes (fluorescent dots) were accumulated due to the GFP-LC3 translocation to the structural components of the double-membrane autophagosome.

Immunofluorescence microscopy

The cells were fixed with 4% paraformaldehyde, permeabilized in 0.5% Triton X-100 and then blocked with 10% goat serum. The cells were incubated for 1 hour with antibodies , and then they were stained for 1 hour with a secondary antibody (1:150). The cells were viewed by fluorescence microscopy.

Supplementary Table 1: The relationship between miR-410-3p expression and clinicopathologic parameters in 86 PDACs

Variable	No of patients	miR-410-3p expression		X2	*P* value
		High	Low		
Age (years)				0.026	0.872
≤60	45	15	30		
>60	41	13	28		
Sex				0.104	0.747
Men	39	12	27		
Women	47	16	31		
Tumor site				0.054	0.816
head and neck	60	20	40		
Body and tail	26	8	18		
Tumor size (cm)				0.219	0.640
≤4	70	22	48		
>4	16	6	10		
Degree of differentiation				0.113	0.737
High and moderate	50	17	33		
Low	36	11	25		
Pt category				1.140	0.286
T1, T2	33	13	20		
T3, T4	53	15	38		
Lymph node metastasis				0.013	0.911
No	56	18	38		
Yes	30	10	20		

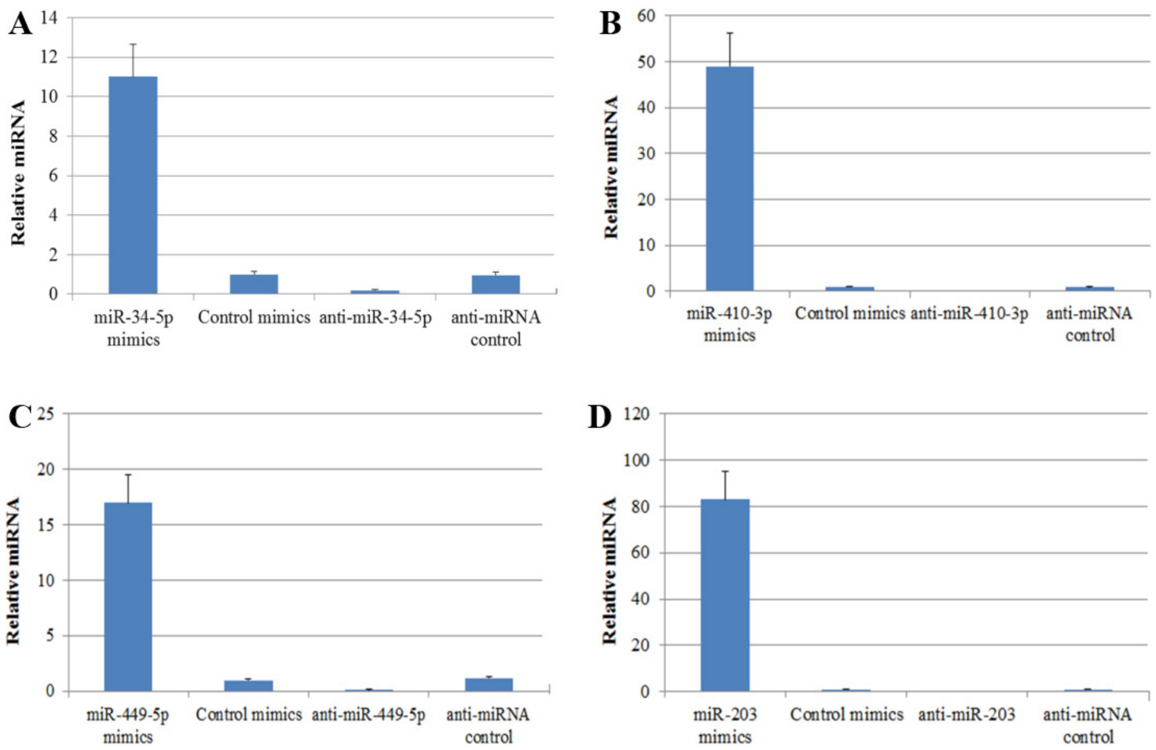

Supplementary Figure 1: miR-34-5p mimics and anti-miR-34-5p, miR-410-3p and anti-miR-410-3p, miR-449-5p and anti-miR-449-5p, or miR-203 and anti-miR-203 was introduced into gemcitabine-resistant PDAC cells. Then the expression of miR-34-5p (**A**), miR-410-3p (**B**), miR-449-5p (**C**) and miR-203 (**D**) was determined by Real-time PCR.

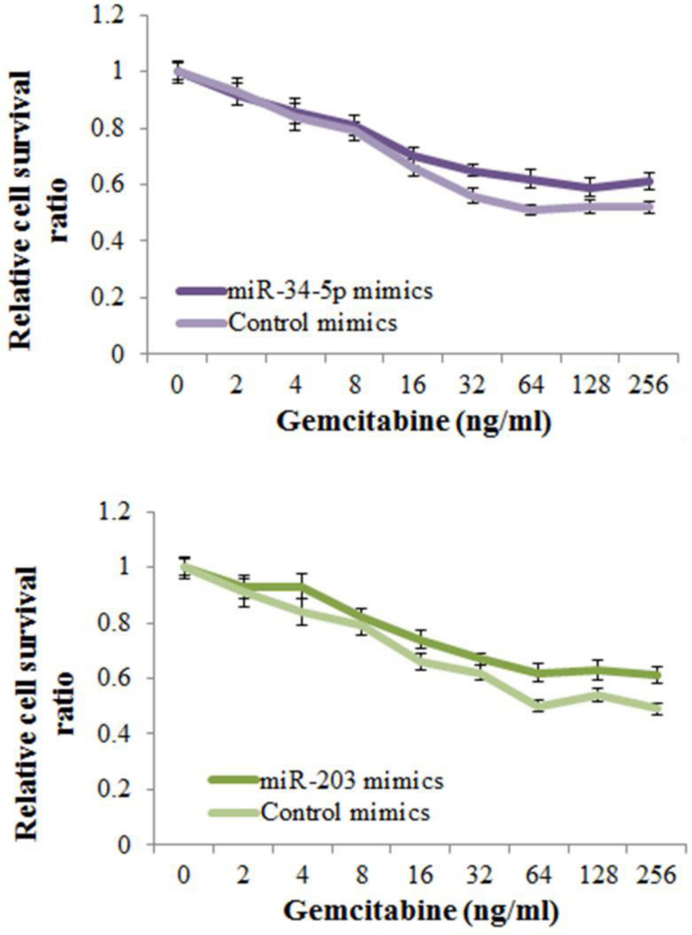

Supplementary Figure 2: Growth-inhibitory effects of gemcitabine on gemcitabine-resistant clones of human PDAC cells treatment with miR-34-5p mimics or miR-203 mimics were assessed by MTT assay.

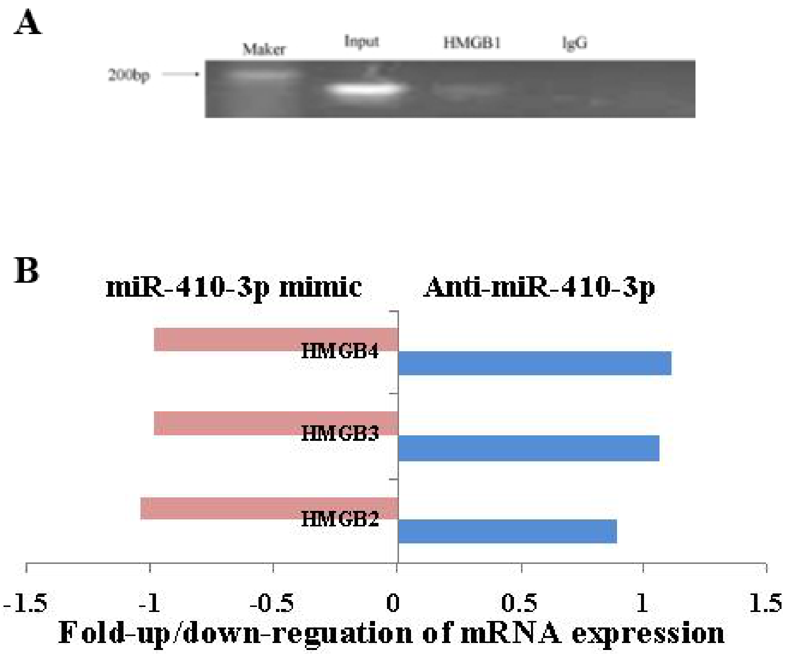

Supplementary Figure 3: (**A**) To confirm the direct interaction between miR-410-3p and HMGB1, ChIP assay was performed in human PDAC cells. The results demonstrated that the protein/DNA complexes precipitated with anti-HMGB1 antibody resulted in a specific PCR product flanking the core sequence. (**B**) The expression of HMGB2, HMGB3 and HMGB4 was determined by Real-time PCR in human PDAC cells transfected miR-410-3p mimic or anti-miR-410-3p.
